# Predicting the global potential distribution of two major vectors of Rocky Mountain Spotted Fever under conditions of global climate change

**DOI:** 10.1371/journal.pntd.0011883

**Published:** 2024-01-10

**Authors:** Haoqiang Ji, Xiaohui Wei, Delong Ma, Xiaoxu Wang, Qiyong Liu

**Affiliations:** 1 Department of Vector Control, Department of Epidemiology, School of Public Health, Cheeloo College of Medicine, Shandong University, Jinan, Shandong province, China; 2 National Key Laboratory of Intelligent Tracking and Forecasting for Infectious Diseases, National Institute for Communicable Disease Control and Prevention, Chinese Center for Disease Control and Prevention; WHO Collaborating Centre for Vector Surveillance and Management, Beijing, China; 3 School of Public Health, Nanjing Medical University, Nanjing, Jiangsu province, China; 4 Jinan Shizhong District Center for Disease Control and Prevention, Jinan, Shandong province, China; Chengde Medical University, CHINA

## Abstract

Rocky Mountain spotted fever is a tick-borne disease that is highly dangerous but often overlooked by the public. To prevent the spread of the disease, it is important to understand the distribution patterns of its vectors’ suitable areas. This study aims to explore the potential global suitability of areas for the vectors of Rocky Mountain spotted fever, including *Dermacentor variabilis* and *Amblyomma cajennense* under both historical and future climate scenarios. The study also seeks to investigate the impact of climatic factors on the distribution patterns of these vectors. Data on species distribution were downloaded from the Global Biodiversity Information Facility, Web of Science and PubMed database. The climatic variables were downloaded from WorldClim Global Climate Database. The Maximum Entropy Model was used to evaluate the contribution of monthly precipitation, monthly maximum temperature, monthly minimum temperature, elevation, and nineteen other climatic variables to vector survival, as well as to predict the suitable area for the vectors. We found that *D*. *variabilis* is distributed in North America, while *A*. *cajennense* is mainly distributed in South America, but all other continents except Antarctica have a suitable distribution. *D*. *variabilis* is more likely to survive in temperate regions, and *A*. *cajennense* is more likely to survive in tropical zones. *D*. *variabilis* is more sensitive to temperature, whereas *A*. *cajennense* is sensitive to both temperature and precipitation, and *A*. *cajennense* prefers tropical regions with hot and humid characteristics. The high suitable areas of both vectors were almost expanded in the ssp5-8.5 scenario, but not so much in the ssp1-2.6 scenario. Highly suitable areas with vectors survival should be strengthened with additional testing to prevent related diseases from occurring, and other highly suitable areas should be alert for entry and exit monitoring to prevent invasion and colonization of vectors.

## Author summary

Rocky Mountain spotted fever is a tick-borne disease that is highly dangerous but often overlooked among the public. For the areas where vector survival has not yet been detected, it is important to understand the distribution patterns of suitable habitats for the vectors in order to proactively prevent their intrusion. However, the global distribution of its vectors (*D*. *variabilis* and *A*. *cajennense*) in their suitable areas has not been fully explored, while the relationship between climatic factors and vectors survival has not been clarified. In this study, we found a wide range of potential habitats for both vectors globally. *D*. *variabilis* is more likely to survive in temperate regions, and *A*. *cajennense* is more likely to survive in tropical zones. *D*. *variabilis* is more sensitive to temperature, whereas *A*. *cajennense* is sensitive to both temperature and precipitation, and *A*. *cajennense* prefers tropical regions with hot and humid characteristics. The highly suitable areas of both vectors were almost expanded in the ssp5-8.5 scenario, but not so much in the ssp1-2.6 scenario. This study highlighted that areas with high suitability should strengthen vector surveillance and public health education to prevent tick bites, and other highly suitable areas should be alert for entry and exit monitoring to prevent invasion and colonization of vectors.

## Introduction

Rocky Mountain spotted fever (RMSF) is one of the most virulent infectious diseases and the most common tick-borne rickettsial disease in the United States [1,2]. In South America, RMSF is also known as Brazilian spotted fever. The pathogen responsible for RMSF was first isolated by Howard T Ricketts but he died after contracting this pathogen [3]. In honor of his contribution, the pathogen was named *Rickettsia rickettsii* and is characterized by high virulence, rapid onset, and high mortality [4,5]. In the early stages of RMSF, patients present with symptoms such as high fever, severe headache, and edema. Delayed treatment can lead to serious consequences such as coma, respiratory failure, and multi-organ system damage [6]. In the past, the mortality rate in untreated patients used to be as high as 87%, but it has decreased to less than 5% after treatment with tetracycline antibiotics [1]. The high incidence of RMSF coincides with the season of tick activity, mainly from April to September [1,7]. Although RMSF was initially endemic only in the United States, host migration or population movement caused the disease to spread northward to northern Canada and southward to southern Brazil [6].

The spread of diseases and vectors is also linked to climate change, which not only affects the migration routes of hosts, but also alters the suitable areas of vectors. Ticks are ectothermic organisms, which means that they rely on ambient temperature or host temperature to determine their activity. Climate and weather sensitivities include survival of ticks and lengthening the duration of the tick activity season. These sensitivities mean that, in some areas, a warmer climate may increase the duration of development and host-seeking activity [8]. These phenomena may increase the chances of vector-human contact and risk of related diseases [9].

Therefore, it is important to explore the changes in the suitable areas of vectors of RMSF driven by climate change in order to effectively prevent their intrusion, and to explore the relationship between climate variables and the probability of vector survival. In North America, the vectors of RMSF are mainly *D*. *variabilis*, *D*. *andersoni* and *Rhipicephalus sanguineus*, while in Central and South America they are *A*. *cajennense* and *A*. *aureolatum*. Additionally, *D*. *variabilis* is the predominant vector found on pets throughout the United States, with its hosts mainly being rodents and small mammals such as cats and dogs [2,10]. The *A*. *cajennense* tick has been thought to be the main vector of RMSF in Central and South America, with capybaras as its main host [10–13]. These two species of ticks can not only transmit RMSF but also carry a variety of pathogens that can lead to epidemics of other diseases, such as *Francisella tularensis*, *Coxiella burnetii*, and *Anaplasma marginale* [14]. In this study, only these two important vectors of RMSF were included to analyze their globally suitable areas and to analyze the relationship between climate factors and species distribution.

## Method

### Species distribution

Data of species’ distribution were downloaded from the Global Biodiversity Information Facility (GBIF; https://www.gbif.org/, accessed on 13 January 2023). (S1 Dataset) In addition, we implemented a literature review to complete the occurrence data from Web of Science and PubMed database, for which the time range was 1970–2020 [11,12,15–25]. These distribution points were filtered to remove duplicates and missing records. The base layer of the world map used in this study were derived from http://www.naturalearthdata.com. We utilized the INSIDE operation to exclude distribution points located in the ocean using ArcGIS software (version 10.6, ESRI Inc., USA). To reduce the spatial sampling deviation and overfitting of models, we used ENMTools to filter the occurrence data so that only one point is kept per grid cell (5 arcmin spatial resolution) [26]. After the above process, we obtained 2455 points for *D*. *variabilis* and 184 points for *A*. *cajennense*. The search terms, inclusion and exclusion criteria are detailed in the S1 Text.

### Climatic variables

The selection of climatic variables mainly depends on their effects on species distribution and the spatial correlation among variables. In the study, the climatic variables of historical climate data (at a 5 arcmin spatial resolution), including monthly precipitation, monthly maximum temperature, monthly minimum temperature, elevation, and nineteen climatic variables from 1970–2000 were downloaded from WorldClim version 2.1 Global Climate Database (http://worldclim.org/version2, accessed on: 13 January 2023). Meanwhile, the future climate conditions were processed using the medium-resolution National (Beijing) Climate Center Climate System Model (BCC-CSM2-MR model) under two greenhouse gas emission scenarios: Shared Socio-economic Pathways (SSPs) ssp1-2.6 and ssp5-8.5, which were acquired from the WorldClim with the 5 arcmin spatial resolution for the years 2021–2100.

To avoid collinearity, some climatic variables need to be removed. We removed climatic variables in two steps. First, Maximum Entropy Model was used to predict climatic variables on species distribution, and we retained climatic variables with a higher contribution rate. Second, ArcGIS was used to sample climatic variables, and R was used to conduct Pearson correlation analysis on retained climatic variables. For climatic variables with correlation coefficient greater than 0.8, only one climatic variables with the highest contribution degree is retained.

### Selection and optimization of model parameters

All distribution points of *D*. *variabilis* and *A*. *cajennense* and the climatic variables were imported into the MaxEnt version 3.4.4, which was set to random seed 75% distribution points modeling and 25% points verification modeling. The output format was “Cloglog”, the maximum iteration mode was set to select “Bootstrap” and the maximum number of repetitions was 5000. Moreover, to get the best model and control overparameterization, the R package “ENMeval” (R 4.1.0 “Checkerboard”) was used to select the best parameters from 5 feature combination (including automatic linear, L; quadratic, Q; hinge, H; product, P; threshold, T) and 8 level regularization multiplier (including 0.5, 1, 1.5, 2, 2.5, 3, 3.5 and 4).

### Distribution and change of suitable area

Used the Maximum Entropy Model in MaxEnt software, the distribution points, climatic variables and model parameters retained by the above operations were used to predict historical and future suitable areas of *D*. *variabilis* and *A*. *cajennense*. Based on log-log (cloglog) values, we used the “natural break point classification (Jenks)” method in ArcGIS to classify four suitable areas for the two species: unsuitability (0–0.099), low suitability (0.099–0.299), moderate suitability (0.299–0.599) and high suitability (0.599–1). Finally, we used ArcGIS to count and calculate the area of each suitable location. The ROC curve was used to test the accuracy of results, and the AUC value was used to show the prediction effect of the model and the correlation between climatic variables and species distribution. The evaluation standard of the ROC curve is: AUC value = 0~0.6, failure; 0.6~0.7, poor; 0.7~0.8, fair; 0.8~1, good.

## Results

### Global distribution of two major vectors of RMSF

**Fig 1:**
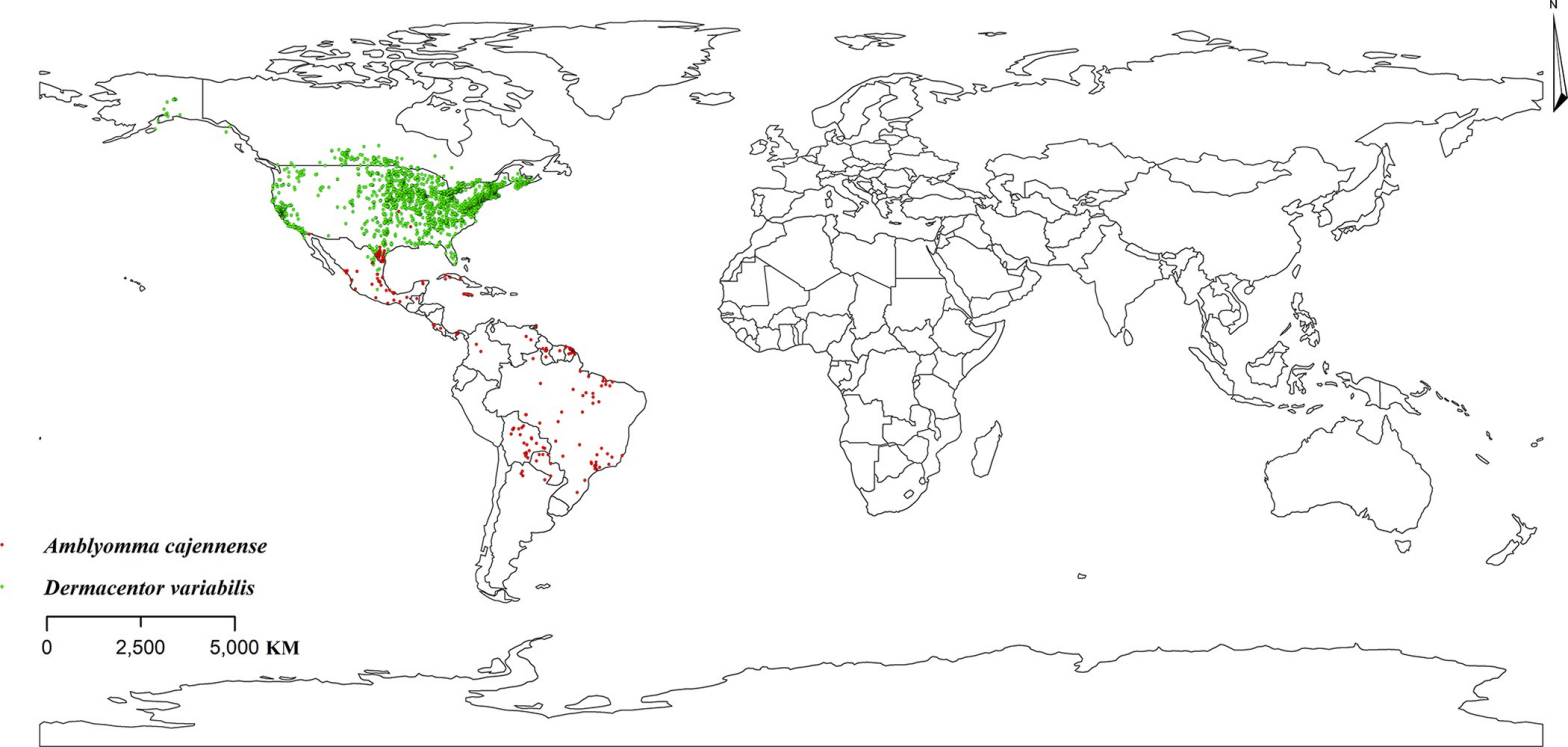
Current global distribution of *D*. *variabilis* and *A*. *cajennense*. The base layer of the map is freely available from http://www.naturalearthdata.com.

After deleting the duplicate points through the ENMTools, we obtained 2,455 occurrence points of *D*. *variabilis* and 184 points of *A*. *cajennense*. *D*. *variabilis* is mainly distributed in most parts of the United States, southern Canada and northern Mexico, and its main survival areas are subtropical and temperate regions. In addition, *A*. *cajennense* is found mainly in Central America and South American countries, such as Brazil and Bolivia, where it survives in tropical rainforest and tropical grassland region (Fig 1).

### The parameters of the Maximum Entropy Model

**Fig 2:**
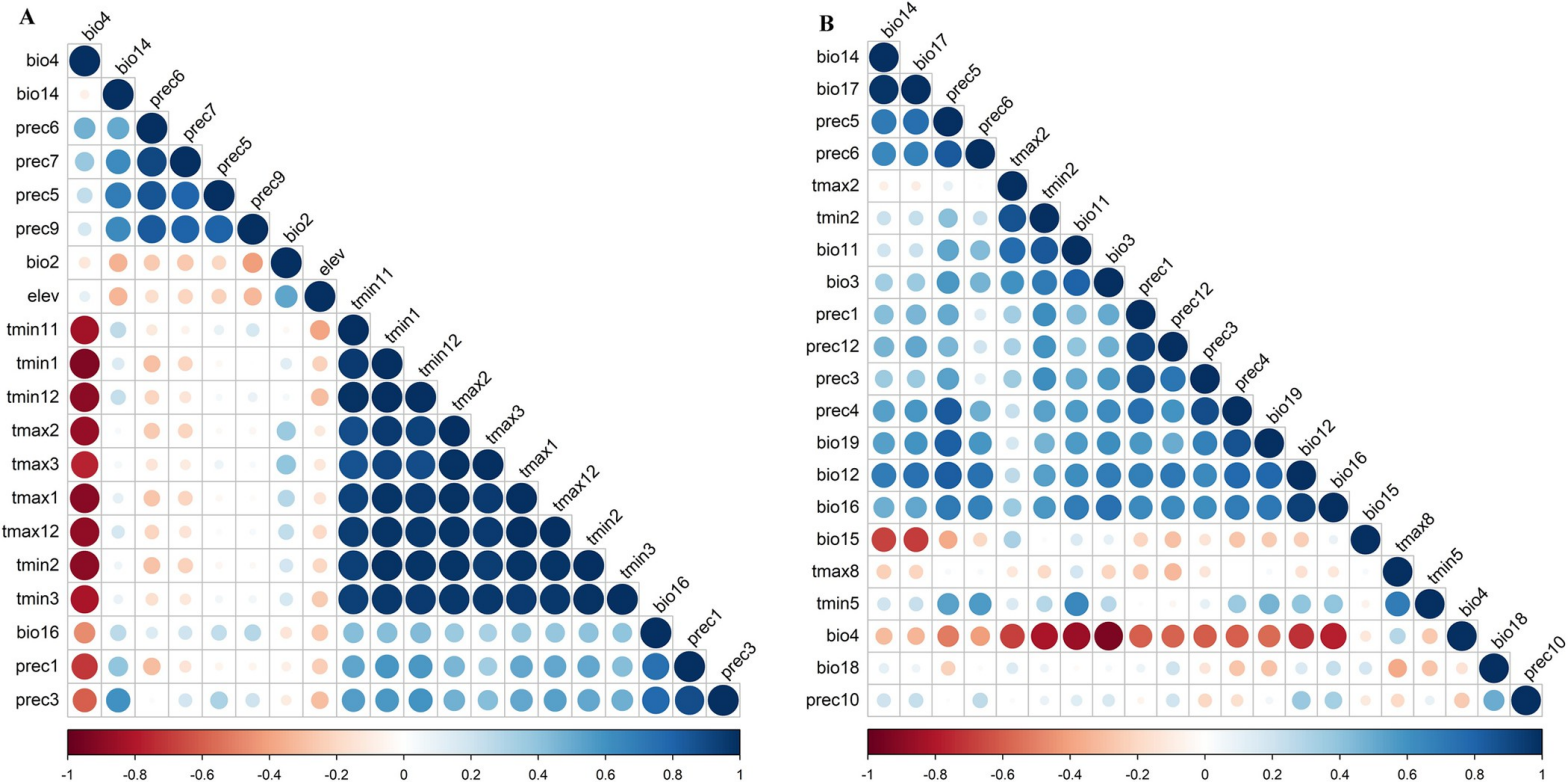
Heat map of Pearson’s correlation coefficient (A: *D*. *variabilis*; B: *A*. *cajennense*).

After the jackknife analysis was applied to retain climatic and environment variables (Fig 2) that contribute rate more than 1% in the Maximum Entropy Model. Pearson analysis was used to analyze the correlation of these climatic variables. Six variables (Table 1) with the highest contribution rates were retained for each species. In addition, the R package “ENMeval” was used to select the best parameters from feature combination (*D*. *variabilis*: “LQHPT”; *A*. *cajennense*: “QHP”) and regularization multiplier (all regularization multipliers were 0.5) when the deltaAICc value was lowest. These parameters and climatic variables were adjusted in the Maximum Entropy models, and the analysis results showed the mean AUC of the MaxEnt models were 0.91 and 0.969 (S1 Fig).

**Table 1:** Climatic variables used for predicting the species distribution.

Variables	Description (Unit)	Contribution (*D*. *variabilis*, %)	Contribution (*A*. *cajennense*, %)
tmax2	Maximum temperature in February (°C)	42.4	26.4
prec6	Precipitation in June (mm)	24.9	-
prec1	Precipitation in January (mm)	14.3	15.3
bio2	Mean diurnal range (°C)	10.9	-
elev	Elevation (m)	6.3	-
bio14	Precipitation of driest month (mm)	1.2	-
bio11	Mean temperature of coldest quarter (°C)	-	22.4
bio12	Annual precipitation (mm)	-	15.3
prec10	Precipitation in October (mm)	-	11.8
bio15	Precipitation seasonality (mm)	-	8.8

### The relationship between climatic variables and the distribution of two major vectors of RMSF

**Fig 3:**
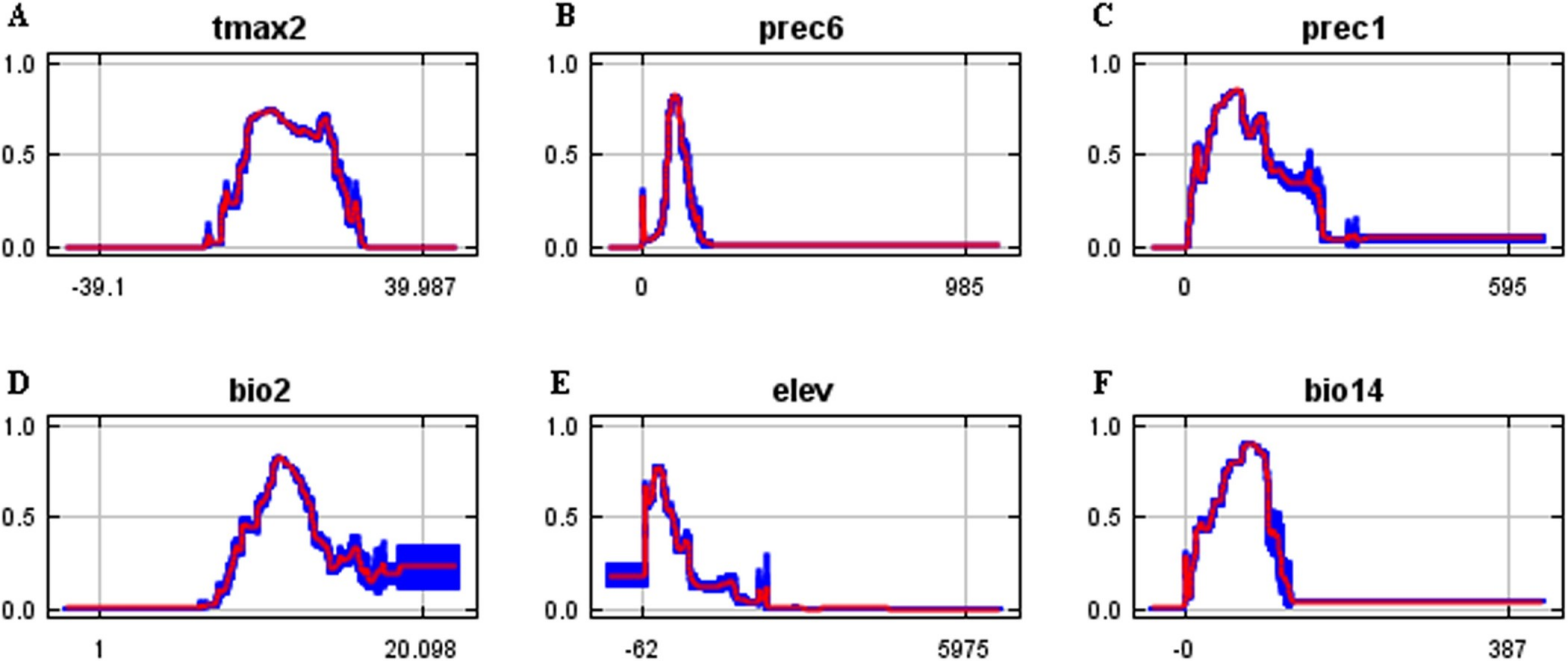
Response curves of climatic variables to the distribution probability of *D*. *variabilis* (y-axis represents survival probability; x-axis unit refers to Table 1).

**Fig 4:**
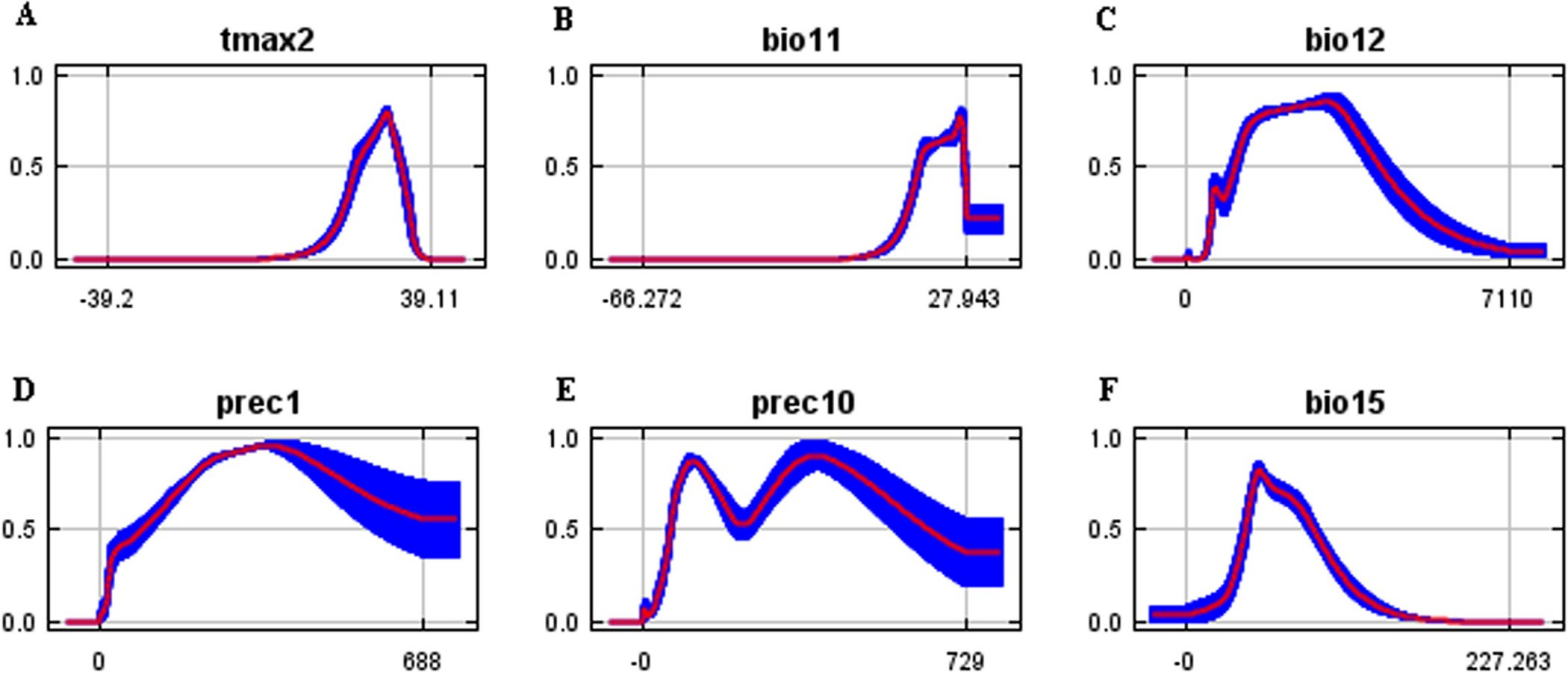
Response curves of climatic variables to the distribution probability of *A*. *cajennense* (y-axis represents survival probability; x-axis unit refers to Table 1).

Temperature seems to have a significant effect on the distribution of *D*. *variabilis*, with the variables maximum temperature in February and mean diurnal range contributing 53.3% to the model, with maximum temperature in February contributing the most (42.4%). All variables showed a roughly inverted U-shaped relationship with species distribution, with the most suitable temperature for *D*. *variabilis* survival at a maximum temperature of 3°C in February, but with a rapidly decreasing trend from 17 to 22°C (Fig 3). However, *A*. *cajennense* prefers a hot and humid environment. Both temperature and precipitation appear to be important for *A*. *cajennense*, but maximum temperature in February and mean temperature of the coldest quarter contribute the most to the model, together accounting for 48%. The maximum temperature in February was 28°C and the mean temperature of the coldest quarter was 27°C, which was the most suitable for *A*. *cajennense* survival. Meanwhile, the annual average precipitation of 3270 mm is the most suitable level for *A*. *cajennense* survival (Fig 4).

### The potential distribution of two major vectors of RMSF under different climate scenarios

**Fig 5:**
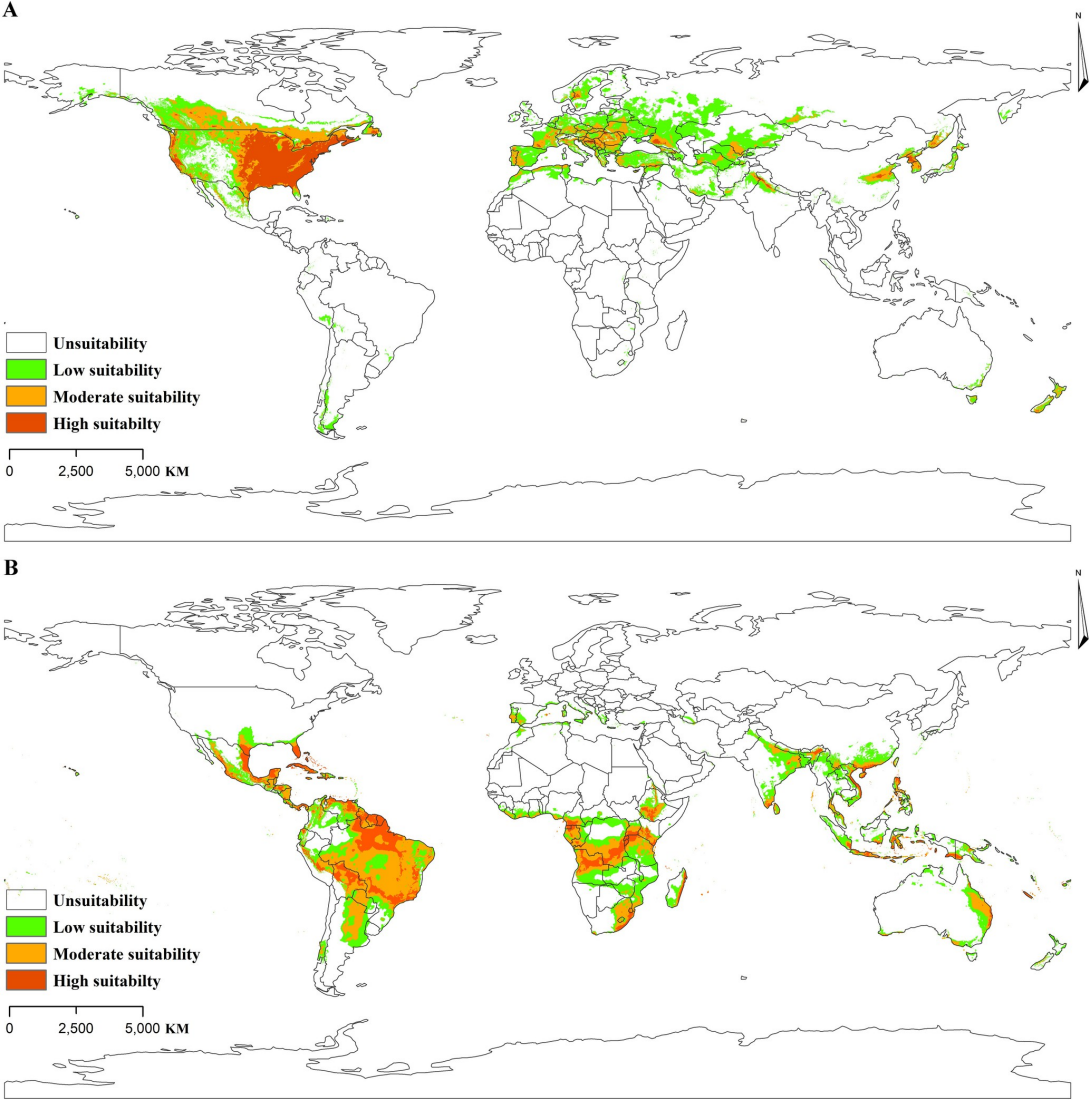
Potentially suitable areas under historical situation (A: *D*. *variabilis*; B: *A*. *cajennense*). The base layer of the map is freely available from http://www.naturalearthdata.com.

Under the historical climate scenarios, suitable areas for *D*. *variabilis* are mainly located in North America, most of Europe, West Asia and a few parts of East Asia. The high suitable area is primarily located in the United States, with a few parts of Europe and East Asia (Fig 5A). Similarly, suitable areas for *A*. *cajennense* are primarily concentrated in tropical regions of Latin America, South America, central Africa, and Southeast Asia (Fig 5B).

Under the different future climate scenarios (ssp1-2.6 and ssp5-5.8), the suitable areas of *D*. *variabilis* showed a decreasing trend (Table 2). Although the total suitable area decreased less under the ssp5-5.8 scenario, the high suitable areas were greater than those seen in the historical and ssp1-2.6 scenarios (S2 and S3 Figs). The maximum suitable was observed to be 44.36×10^6^ km^2^ between 2021–2040 under the ssp5-8.5 scenario, while the minimum suitable area was 40.08 ×10^6^ km^2^ between 2061–2080 under the ssp1-2.6 scenario. In addition, all total suitable areas of *A*. *cajennense* expanded between 2021–2040 when compared to the historical scenario (S4 and S5 Figs). The largest total suitable areas and high areas were observed between 2021–2040 under the ssp5-8.5 scenario, with a total suitable area of 41.88 ×10^6^ km^2^ and a high area of 9.85 ×10^6^ km^2^, which was greater than other scenarios as noted in Table 2.

**Table 2:** Suitable areas for vectors of RMSF across the world under different climatic scenarios (×10^6^ km^2^).

Climate Scenarios	Period	Less suitability	Moderately suitability	High suitability	Total area	Area change	Area change Rate (%)
*D*. *variabilis*							
Historical	1970–2000	26.15	12.76	7.03	45.94	-	-
ssp1-2.6	2021–2040	20.67	14.53	6.27	41.46	-4.47	-9.74
	2041–2060	19.60	15.04	6.65	41.29	-4.64	-10.11
	2061–2080	18.93	14.14	7.01	40.08	-5.85	-12.74
	2081–2100	19.68	16.18	7.06	42.92	-3.01	-6.56
ssp5-8.5	2021–2040	22.38	13.92	8.06	44.36	-1.57	-3.42
	2041–2060	20.92	12.93	7.11	40.96	-4.98	-10.84
	2061–2080	20.23	12.99	7.00	40.23	-5.71	-12.43
	2081–2100	22.79	12.85	7.32	42.96	-2.98	-6.49
*A*. *cajennense*							
Historical	1970–2000	15.37	14.11	6.21	35.68	-	-
ssp1-2.6	2021–2040	15.73	14.50	7.83	38.06	2.38	6.67
	2041–2060	13.95	12.34	5.64	31.93	-3.75	-10.51
	2061–2080	14.78	11.12	5.84	31.74	-3.94	-11.03
	2081–2100	15.57	11.12	8.74	35.43	-0.25	-0.70
ssp5-8.5	2021–2040	17.34	14.68	9.85	41.88	6.20	17.38
	2041–2060	14.34	10.92	6.44	31.70	-3.98	-11.16
	2061–2080	14.51	11.41	5.89	31.81	-3.87	-10.85
	2081–2100	15.75	13.90	8.50	38.16	2.48	6.94

## Discussion

In this study, we found that the two vectors of RMSF were distributed only in the Americas, but all other continents except Antarctica had a suitable distribution. While *D*. *variabilis* is more likely to survive in temperate regions, *A*. *cajennense* is more likely to survive in tropical regions [27]. *D*. *variabilis* is more sensitive to temperature, and neither tropical nor boreal regions are suitable for its survival. *A*. *cajennense* is sensitive to both temperature and precipitation, and prefers tropical regions with high temperature and hot and humid characteristics. The high suitable areas of both vectors were almost expanded in the ssp5-8.5 scenario, but not so much in the ssp1-2.6 scenario.

Vector densities may be higher in high suitable areas, which could increase the population’s exposure to vectors and the risk of infectious disease [9]. This should alert areas with vector colonization and increase local health education campaigns. Meanwhile, monitoring of vector densities in high suitable areas should be enhanced during the tick activity season so that effective measures can be taken to prevent the occurrence of related diseases. The public is reminded that when keeping pets or playing outdoors, they should regularly check their skin for tick bites and wear long clothing or take other measures to prevent tick bites. If a local vector is found to be colonizing the area, targeted screening or treatment should be administered after the patient has been bitten, thus saving medical costs and preventing delayed treatment. In addition, for those Asian and European countries with highly suitable areas, the entry and exit quarantine capacity should be strengthened during trade, pet transportation or population movement with natural epidemic foci to prevent vector invasion and colonization in local areas. It is worth noting that *D*. *variabilis* corresponds to two species, among which *D*. *similis* n. sp. has minor morphological differences compared to *D*. *variabilis* and is primarily distributed in the western regions of the Rocky Mountains [28]. This reminds relevant departments to pay closer attention when identifying ticks originating from this area. Moreover, travelers with a history of traveling to high-risk areas should promptly check themselves, pets, and belongings for the presence of ticks upon the completing their trip, which can effectively prevent cross-regional transmission of the vector. Such cases existed in the past. For example, when a woman returned from a trip to the United States, she found a tick bite on his arm, which was the first time that *D*. *variabilis* was found to exist in Europe [29]. Moreover, a tourist with a history of travel to the United States also found *D*. *variabilis* on her pet dog [30]. There is no evidence to prove that the species has settled in the area due to either timely detection or unsuitable climate for its survival. However, entry and exit quarantine departments in suitable areas should not let their guard down in the future. With increasing international exchanges, the invasion and colonization of these two species may pose serious health threats.

The circulation of a pathogen and subsequent transmission to humans is rather intricate interplay between vector, pathogen, reservoir and human behaviour. The diversity and density of vectors are related to ecological environment and climate change [31]. In this study, temperature was the most influential factor in determining the survival of both vectors, which may be related to the fact that ticks need a certain temperature to survive in winter. These findings are consistent with F. Strey’s study [27] which suggests that *A*. *cajennense* is less resistant to desiccation and cold. *A*. *cajennense* has the highest survival rate at a temperature of 23°C with 85% humidity, and its mortality gradually increases with decreasing humidity and temperature [27]. However, *D*. *variabilis* is more resistant to cold and can still survive in the coldest season at 3°C, making it better adapted to temperate climate regions. The density of vector organisms may be higher in areas where the climate is suitable for vector survival, leading to the occurrence of RMSF. In the ssp5-8.5 scenario, there is a tendency for the high suitable areas of both vectors to expand in future stages. This expansion may be related to the highest global warming in this scenario. As the global climate warms, the habitat of ticks may gradually spread to colder parts of the Earth’s poles [32]. Governments should pay attention to greenhouse gas emissions and make efforts to avoid global warming and extreme weather events. Such efforts play an important role in preventing the expansion of suitable areas.

There are still some limitations in this study. Firstly, many other factors affect vector survival, such as vegetation area, soil moisture, host density, and other factors not included in the model. Secondly, due to the limited literature search, not all species distribution points were searched, which may have some influenced the estimation results of the model. Thirdly, this study only presents a few possible modes of vector invasion, and the specific invasion methods and associated risks should be further explored in future research. Finally, *D*. *variabilis* and *A*. *cajennense* are not the only two vectors of RMSF, and the global distribution of other important ticks should be explored in the future.

## Conclusion

In this study, we found that the two vectors of RMSF have potential habitat areas in all continents except Antarctica. While *D*. *variabilis* is more likely to survive in temperate regions, *A*. *cajennense* is more likely to survive in tropical zones. The high suitable areas of both vectors were almost expanded considerably in the ssp5-8.5 scenario but not as much in the ssp1-2.6 scenario. With increased international exchange, vectors of RMSF may have more opportunities to invade other countries. This highlights the importance of raising awareness and alert about the risk areas of vector invasion.

## Supporting information

S1 Text Literature search terms, search details and inclusion and exclusion criteria for this study. (DOCX)

S1 Fig The validation of the Maxent models (Fig A: *D*. *variabilis*; Fig B: *A*. *cajennense*).
(DOCX)

S2 Fig Potentially suitable areas of *D*. *variabilis* under the climatic conditions of ssp1-2.6 during different periods of the 21^st^ century (A: 2021–2040; B: 2041–2060; C: 2061–2080; D: 2081–2100). (DOCX)

S3 Fig Potentially suitable areas of *D*. *variabilis* under the climatic conditions of ssp5-8.5 during different periods of the 21^st^ century (A: 2021–2040; B: 2041–2060; C: 2061–2080; D: 2081–2100).
(DOCX)

S4 Fig Potentially suitable areas of *A*. *cajennense* under the climatic conditions of ssp1-2.6 during different periods of the 21^st^ century (A: 2021–2040; B: 2041–2060; C: 2061–2080; D: 2081–2100).
(DOCX)

S5 Fig Potentially suitable areas of *A*. *cajennense* under the climatic conditions of ssp5-8.5 during different periods of the 21^st^ century (A: 2021–2040; B: 2041–2060; C: 2061–2080; D: 2081–2100).
(DOCX)

S1 Dataset Distribution points of two vectors retrieved from the Global Biodiversity Information Facility and other details. (XLSX)
