# Supplementary material for: Predicting the global potential distribution of two major vectors of Rocky Mountain Spotted Fever under conditions of global climate change
Source: PLoS Negl Trop Dis. 2024 Jan 10;18(1):e0011883. doi: 10.1371/journal.pntd.0011883 (PMC10805312; doi:10.1371/journal.pntd.0011883)
Supplement: S1 Text — (DOCX) [file pntd.0011883.s001.docx]

**S1 Text. Literature search terms, search details and inclusion and exclusion criteria for this study**

**Search terms**

(("Amblyomma cajennense") OR ("Cayenne tick")OR ("Dermacentor variabilis") OR ("American dog tick") OR ("Brazilian spotted fever") OR ("Rocky Mountain Spotted Fever") OR ("Rickettsia rickettsii")) AND (distribution)

**Search Details**

("Amblyomma cajennense"[All Fields] OR "Cayenne tick"[All Fields] OR "Dermacentor variabilis"[All Fields] OR "American dog tick"[All Fields] OR "Brazilian spotted fever"[All Fields] OR "Rocky Mountain Spotted Fever"[All Fields] OR "Rickettsia rickettsii"[All Fields]) AND ("distribute"[All Fields] OR "distributed"[All Fields] OR "distributer"[All Fields] OR "distributers"[All Fields] OR "distributes"[All Fields] OR "distributing"[All Fields] OR "distributional"[All Fields] OR "distributions"[All Fields] OR "supply and distribution"[MeSH Subheading] OR ("supply"[All Fields] AND "distribution"[All Fields]) OR "supply and distribution"[All Fields] OR "distribution"[All Fields])

**Inclusion and exclusion criteria**

First, we included all articles in the range 1970-2020 containing "Amblyomma cajennense", "Cayenne tick"," Dermacentor variabilis" and "American dog tick", in our literature database. Next, we removed duplicate articles and those that did not include latitude and longitude information. Subsequently, we added the latitude and longitude coordinates of the distribution points for each of the two species to their respective databases.
