# Supplementary figures and images for: Predicting the global potential distribution of two major vectors of Rocky Mountain Spotted Fever under conditions of global climate change
Source: PLoS Negl Trop Dis. 2024 Jan 10;18(1):e0011883. doi: 10.1371/journal.pntd.0011883 (PMC10805312; doi:10.1371/journal.pntd.0011883)

**Fig S1. The validation of the Maxent models (Fig A: D. variabilis; Fig B: A. cajennense).**


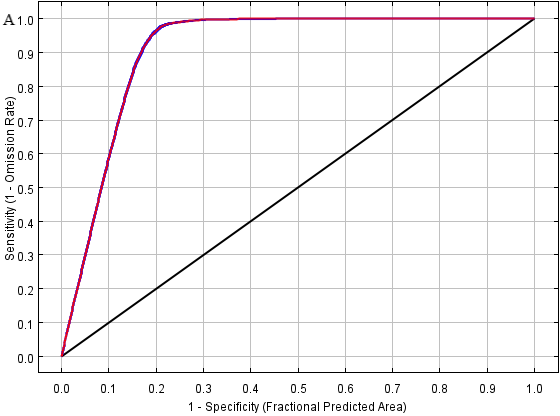

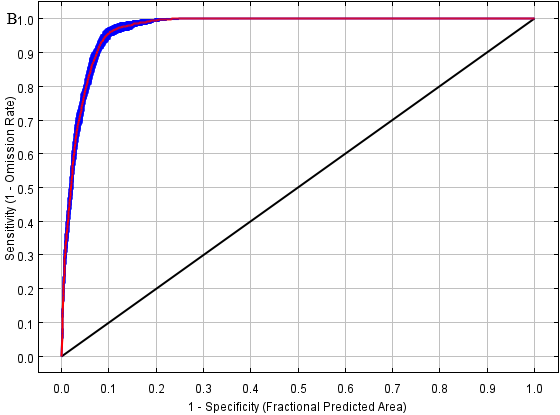

Supplement: S1 Fig — The validation of the Maxent models (Fig A: D. variabilis; Fig B: A. cajennense). (DOCX) [file pntd.0011883.s002.docx]
