# Supplementary material for: Predicting the global potential distribution of two major vectors of Rocky Mountain Spotted Fever under conditions of global climate change
Source: PLoS Negl Trop Dis. 2024 Jan 10;18(1):e0011883. doi: 10.1371/journal.pntd.0011883 (PMC10805312; doi:10.1371/journal.pntd.0011883)

**Fig S3. Potentially suitable areas of D. variabilis under the climatic conditions of ssp****5-8.5 during different periods of the 21st century (A: 2021-2040; B: 2041-2060; C: 2061-2080; D: 2081-2100).** **The base layer of the map is available from http://www.naturalearthdata.com.**


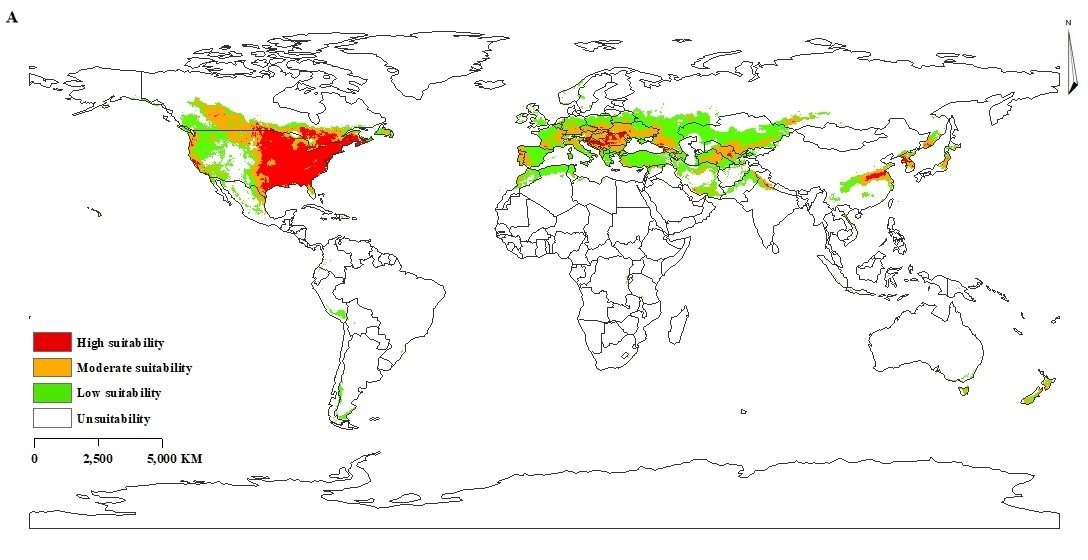

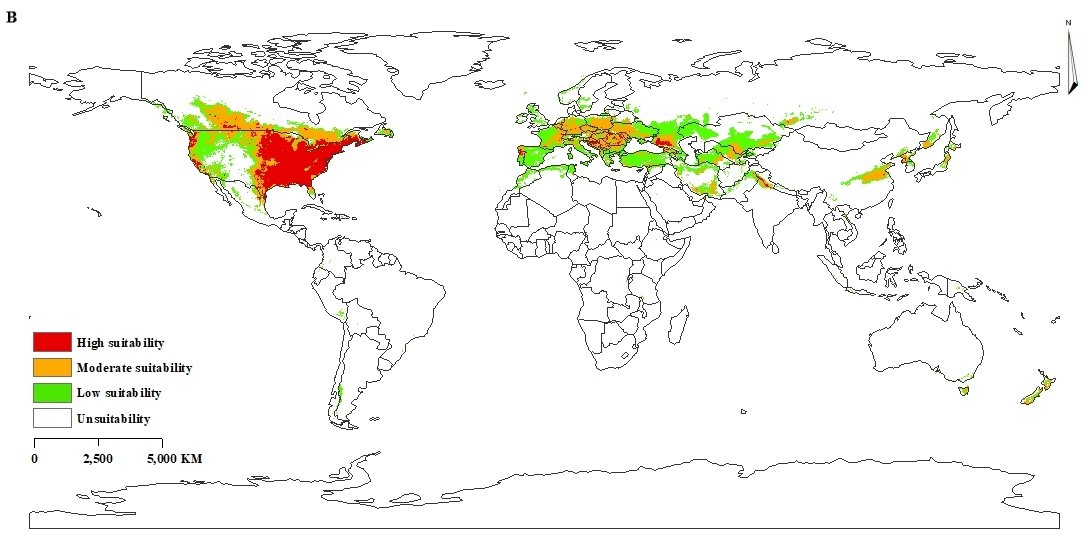

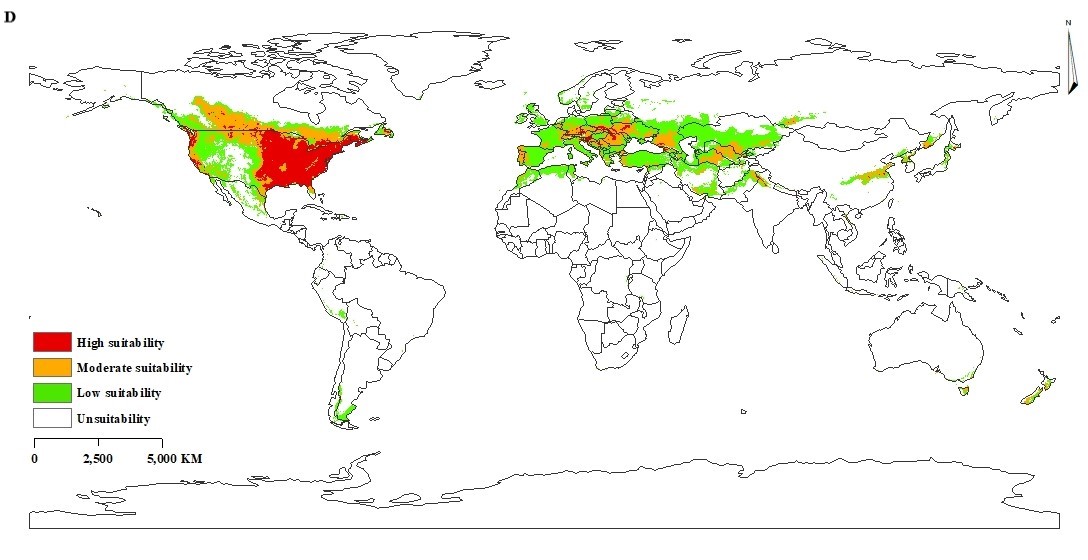

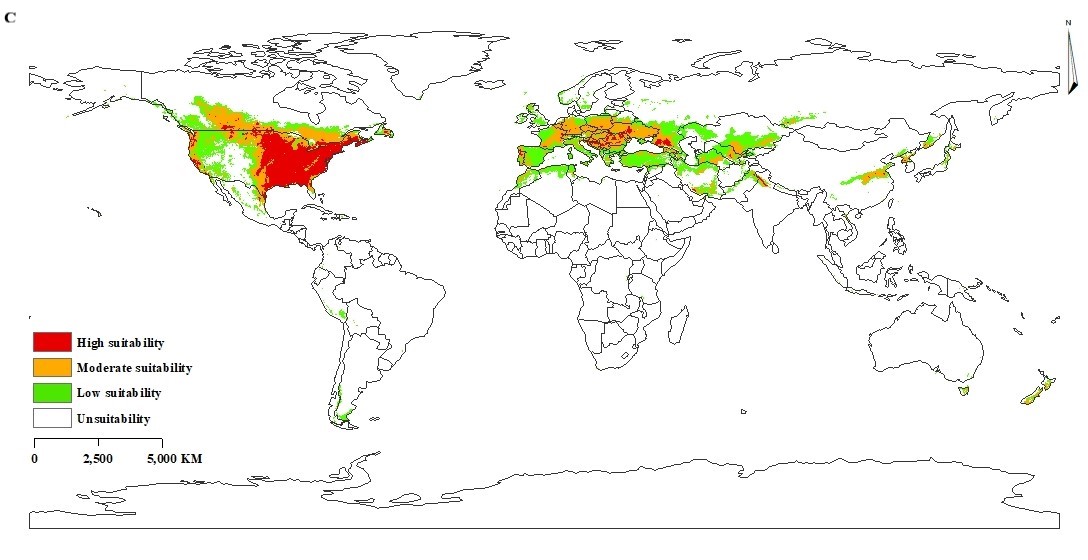

Supplement: S3 Fig — Potentially suitable areas of D. variabilis under the climatic conditions of ssp5-8.5 during different periods of the 21st century (A: 2021–2040; B: 2041–2060; C: 2061–2080; D: 2081–2100). (DOCX) [file pntd.0011883.s004.docx]
